# Expression of Biliverdin Reductase A in Peripheral Blood Leukocytes Is Associated with Treatment Response in HCV-Infected Patients

**DOI:** 10.1371/journal.pone.0057555

**Published:** 2013-03-11

**Authors:** Iva Subhanova, Lucie Muchova, Martin Lenicek, Hendrik J. Vreman, Ondrej Luksan, Kristyna Kubickova, Miluse Kreidlova, Tomas Zima, Libor Vitek, Petr Urbanek

**Affiliations:** 1 Institute of Medical Biochemistry and Laboratory Diagnostics, General University Hospital and 1st Faculty of Medicine, Charles University in Prague, Prague, Czech Republic; 2 Department of Pediatrics, Stanford University School of Medicine, Stanford, California, United States of America; 3 Centre of Experimental Medicine, Institute for Clinical and Experimental Medicine, Prague, Czech Republic; 4 Department of Internal Medicine, 1st Faculty of Medicine, Charles University in Prague, Prague, Czech Republic; 5 4th Department of Internal Medicine, 1st Faculty of Medicine, Charles University in Prague, Prague, Czech Republic; Duke University, United States of America

## Abstract

**Background and Aims:**

Hepatitis C virus (HCV) infection is associated with systemic oxidative stress. Since the heme catabolic pathway plays an important role in antioxidant protection, we attempted to assess the gene expression of key enzymes of heme catabolism, heme oxygenase 1 (HMOX1), heme oxygenase 2 (HMOX2), and biliverdin reductase A (BLVRA) in the liver and peripheral blood leukocytes (PBL) of patients chronically infected with HCV.

**Methods:**

Gene expressions (*HMOX1, HMOX2, BLVRA*) and HCV RNA were analyzed in PBL of HCV treatment naïve patients (n = 58) and controls (n = 55), with a subset of HCV patients having data on hepatic gene expression (n = 35). Based upon the therapeutic outcome, HCV patients were classified as either responders (n = 38) or treatment-failure patients (n = 20). Blood samples in HCV patients were collected at day 0, and week 12, 24, 36, and 48 after the initiation of standard antiviral therapy.

**Results:**

Compared to the controls, substantially increased *BLVRA* expression was detected in PBL (p<0.001) of therapeutically naïve HCV patients. mRNA levels of *BLVRA* in PBL closely correlated with those in liver tissue (r2 = 0.347,p = 0.03). A marked difference in *BLVRA* expression in PBL between the sustained responders and patients with treatment failure was detected at week 0 and during the follow-up (p<0.001). Multivariate analysis revealed that *BLVRA* basal expression in PBL was an independent predictor for sustained virological response (OR 15; 95% CI 1.05–214.2; P = 0.046). *HMOX1/2* expression did not have any effect on the treatment outcome.

**Conclusion:**

Our results suggest that patients with chronic HCV infection significantly upregulate *BLVRA* expression in PBL. The lack of *BLVRA* overexpression is associated with non-responsiveness to standard antiviral therapy; whereas, *HMOX1/2* does not seem to have any predictive potential.

## Introduction

Hepatitis C virus (HCV) infection represents one of the leading causes of chronic hepatitis worldwide, resulting in progression into fibrosis, cirrhosis and hepatocellular carcinoma in a significant number of HCV-infected patients [1]. The HCV prevalence is estimated to be 3% worldwide [2] and 0.2–0.5% in the Czech Republic with predominance of genotype 1 (79.3%) and 3 (19.7%) [3], [4].

Although HCV is mainly hepatotropic, there is also evidence that it can replicate in the peripheral blood mononuclear cells (PBMC) of patients with chronic HCV infection [5].

**Figure 1:**
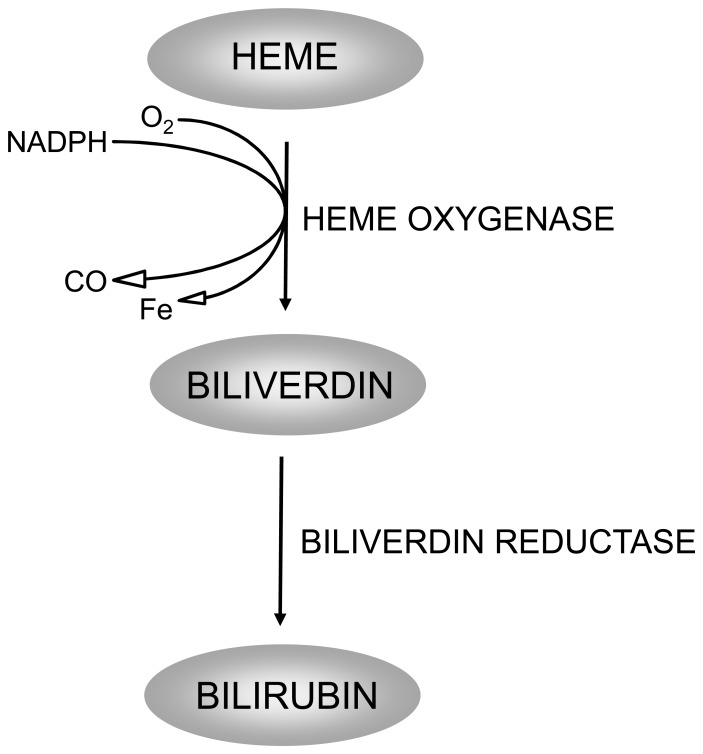
Heme catabolic pathway. Heme oxygenase, the rate limiting enzyme in the heme catabolic pathway, catalyzes oxidative degradation of heme to form equimolar amounts of bioactive products carbon monoxide, iron and biliverdin, which is subsequently reduced to bilirubin by biliverdin reductase.

Oxidative damage has been hypothesized to play a role in HCV-induced liver disease, with reactive oxygen and nitrogen species (RONS) generated from HCV-infected hepatocytes, and infiltrating the immune cells [6], [7]. HCV might not only increase RONS production, but also downregulate expression of certain antioxidant genes, including heme oxygenase (HMOX) [8]. HMOX catalyzes the degradation of the pro-oxidative heme to biliverdin, carbon monoxide (CO), and iron (Figure 1). Biliverdin is then subsequently reduced to bilirubin by biliverdin reductase (BLVR). Biliverdin, bilirubin, and CO exert numerous biological functions, including anti-oxidative and anti-inflammatory effects, as well as the modulation of cell proliferation and apoptosis [9], [10], [11]. Two HMOX isoforms have evolved, which include HMOX1 (OMIM*141250**)**, an inducible isoenzyme, and HMOX2 (OMIM*141251), a constitutive isoform. Both catalyze the same reaction, but are differentially regulated, and play different roles in protecting tissues against oxidative injuries [12]. An accumulating body of evidence suggests that *HMOX1* overexpression contributes to cellular response against oxidative stress [13], and might have strong anti-fibrotic, as well as an anti-apoptotic potential within the liver tissue [14], [15]. *HMOX1* induction *in vitro* has recently been shown to decrease HCV replication [16]. On the other hand, reduced *HMOX1* expression has been reported in the liver tissue of patients with chronic hepatitis C [8]; although under *in vitro* conditions hepatic *HMOX1* overexpression in the presence of HCV proteins has been reported by other authors [17].

BLVR, the other enzyme involved in the heme catabolic pathway, is also implicated in the oxidative stress response [18]. Apart from its antioxidative effects, a cytoprotective action independent of heme degradation has been reported [19], [20]. In fact, BLVR has been demonstrated to affect cell signaling pathways by regulating stress-responsive genes, including both *HMOX1* [21], [22], and *HMOX2* [23]. Two isoforms of human BLVR, BLVRA (OMIM*109750) and BLVRB (OMIM*600941), products of different genes, have been described [24]. *BLVRA,* the major form of BLVR in the human adult liver, is subject to regulation by tumor necrosis factor-α, as well as by oxidative stress or hypoxia [25]. Importantly, biliverdin has been shown to inhibit HCV replication [26]. Lehmann *et al.* [26] recently demonstrated that biliverdin interferes with HCV replication-mediated oxidative stress by inducing the expression of antiviral interferons. Huang et al. [27] reported an association between sustained virological response (SVR) and expression of *BLVRB,* the embryonic form of BLVR in PBMC during the first weeks of antiviral therapy. However, no data are available on *BLVRA* expression in the liver, or PBL in chronic HCV infection.

Therefore, the present study was conducted to evaluate the possible role of HCV infection on *HMOX1/HMOX2/BLVRA* gene expression in the liver and PBL, as well as whether these genes may influence or predict the treatment response.

## Patients and Methods

### Patients

The study was performed on 58 consecutive therapeutically naïve patients with chronic HCV infection. The patients were recruited between 2007–2011 at the Hepatology Center in the Central Military Hospital in Prague, Czech Republic. Patients with positivity of anti-HCV antibodies, and detectable HCV RNA in serum for at least 6 months, were included in the study. Detailed description of patients enrolled is given in Table 1.

**Table 1:** Baseline characteristics of patients with hepatitis C.

	Controls (N = 55)	HCV (N = 58)	SVR (N = 38)	NVR (N = 20)	*p*-value*
Gender (M:F ratio)	0.49	1.2	1.0	1.9	0.28
Age (years)	32.5 (27.8–44.0)	45.0 (38.0–53.8)	43.5 (37.3–51.8)	46.0 (43.5–54.0)	0.22
Viral load (log IU/ml)		6.05±0.67	6.05±0.75	6.05±0.50	0.98
Genotype HCV 1 (%)		84	76	94	0.20
*IL28B*(rs12979860)CC(%)		37	44	22	0.11
HMOX activity PBMC	36.3±18.1	20.6±16. 3 (N = 53)	19.1±17.6 (N = 34)	22.7±19.3 (N = 19)	0.47
*HMOX1* expression PBL	4.0±0.89	3.45±2.03	3.63±1.91	3.15±2.31	0.71
*HMOX2* expression PBL	4.98±0.94	4.30±1.13	4.22±0.97	4.41±1.41	0.72
*BLVRA* expression PBL	1.28±0.36	1.68±0.68	1.87±0.74	1.32±0.34	**<0.001**
Liver histology grading		5(4–5)	5(3–5)	4.5(4–6)	0.36
Liver histology staging		1(1–3)	1(1–2)	3.0(1–5)	**0.03**
Total bilirubin (µmol/l)		15.9 (12,4–19.4)	15.0 (11.4–20.6)	16.7 (14.6–18.3)	0.66
ALT (µkat/l)		0.97 (0.77–1.80)	0.93 (0.76–1.39)	1.08 (0.85–2.19)	0.95
AST (µkat/l)		0.67 (0.53–1.21)	0.63 (0.49–1.25)	0.79 (0.58–1.13)	0.71
ALP (µkat/l)		1.28 (0.99–1.95)	1.28 (1.05–2.02)	1.32 (0.99–1.83)	0.19
GGT (µkat/l)		0.68 (0.49–1.76)	0.57 (0.41–1.69)	1.15 (0.66–1.96)	0.73
Hemoglobin (g/l)		146.9±16.6	143.5±17	153.4±14.1	**0.02**
Platelets (x 10^9^/l)		195.5±63.4	205.5±66.4	177.0±54.1	0.08

HCV, hepatitis C virus; PBMC, peripheral blood mononuclear cells; PBL, peripheral blood leukocytes, SVR, responders; NVR, non-SVR patients; HMOX1, 2, heme oxygenase 1, 2; BLVRA, biliverdin reductase A; ALT, serum alanine aminotransferase; ALP, alkaline phosphatase; GGT, gamma glutamyl transpeptidase; N = number of patients. HMOX activity expressed as pmol CO/hr/10^6^ cells. Data expressed as mean±standard deviation (SD), or median (IQ range). *P-value calculated between SVR and NVR.

The patients received standard antiviral therapy (pegylated interferon alpha in combination with ribavirin (PEG-IFN-alpha/RBV)) according to EASL and AASLD practice guidelines [28]. The treatment regimens were: 1) PEG-IFN alpha 2a (Pegasys; Roche, Basel, Switzerland): 180 µg once weekly+RBV (Copegus; Roche, Basel, Switzerland ) 1000–1200 mg daily, according to body weight (1000 mg ≤75 kg, 1200 mg >75 kg); and 2) PEG-IFN alpha-2b (PegIntron; Schering-Plough, Kenilworth, NJ, USA): 1.5 µg/kg body weight once weekly+RBV (Rebetol; Schering-Plough, Kenilworth, NJ, USA): 1000–1200 mg daily, according to body weight (1000 mg ≤75 kg, 1200 mg >75 kg).

The total duration of the antiviral therapy was defined by the type of antiviral response within the first 12–24 weeks of therapy. Patients with HCV RNA decrease <2 log from baseline level at week 12 were classified as null responders and their antiviral therapy was terminated at week 12. Patients with HCV RNA decrease ≥2 log or with undetectable HCV RNA at week 12 were treated to week 24. If HCV RNA was detectable at week 24, patients were classified as slow responders and their therapy was terminated at week 24. Only patients with undetectable HCV RNA at week 24 were treated up to week 48.

Based on the treatment response, patients were classified into two groups. Responders were defined as patients with sustained virologic response (SVR, undetectable HCV RNA at weeks 24 after completion of antiviral therapy, n = 38). Treatment-failure patients (non-SVR, n = 20) included those who did not achieved SVR (n = 15) and patients who relapsed (n = 5).

For PBMC and PBL studies, 55 healthy volunteers (blood donors or employees of General Faculty Hospital and 1^st^ Faculty of Medicine, Charles University in Prague) were used as control subjects. The study was registered under ID: NCT 00842250 (www.clinicaltrial.gov).

The study protocol conformed to all ethical guidelines of the 1975 Declaration of Helsinki, reflected in the *a priori* approval by the institution’s Ethics Committee. Additionally, all subjects in this study had provided written informed consent.

### Methods

#### Material sampling and storage

Patients’ blood samples were collected on the day before treatment initiation (day 0, n = 58), as well as at 12 (n = 37), 24 (n = 31), 36 (n = 27) and 48 (n = 16) weeks after initiation of the standard antiviral treatment. Samples for gene expression analyses were collected into PAXgene Blood RNA Tubes (PreAnalytix, Hombrechtikon, Switzerland) and stored at −80°C. Samples for determination of total HMOX activity in PBMC were collected in BD Vacutainer Blood collection tubes with heparin (BD Diagnostics-Preanalytical Systems, Franklin Lakes, NJ, USA). PBMC were isolated through a Ficoll-density gradient within 5 hours, and stored in potassium phosphate buffer at −80°C. The blood samples for analysis of interleukin 28B (*IL28B,* OMIM*607402*)* gene polymorphism were collected in BD Vacutainer Blood collection tubes with EDTA (BD Diagnostics-Preanalytical Systems, Franklin Lakes, NJ, USA) and stored at −80°C.

All liver samples were immediately placed into RNAlater (Ambion Diagnostics, Austin, TX, USA) and stored at −80°C until total RNA isolation.

#### Reagents

Hemin, nicotinamide adenine dinucleotide phosphate (NADPH) and sulfosalicylic acid were purchased from Sigma (St. Louis, MO, USA). Sodium dihydrogen phosphate (NaH_2_PO_4_) and disodium hydrogen phosphate (Na_2_HPO_4_) were of analytical grade, and purchased from Penta (Prague, Czech Republic).

#### Markers of liver injury

Liver biopsy samples were evaluated according to the Ishak scoring system [29]. Serum markers of liver injury (ALT, AST, GGT, ALP) and bilirubin were analyzed by routine assays on an automated analyzer (Modular analyzer, Roche Diagnostics GmbH, Mannheim, Germany).

#### Serological analyses

HCV RNA in sera was quantified using Roche Cobas AmpliPrep/Cobas TaqMan (detection limit of 15 IU/ml) (Roche Diagnostics GmbH, Mannheim, Germany). HCV genotypes were analysed with VERSANT HCV Genotype 2.0 Assay (LiPA) (Siemens Healthcare Diagnostics, Camberley, UK).

#### Genomic DNA isolation and *IL28B* genotyping

Genomic DNA was extracted from EDTA coagulated peripheral blood using MagNA Pure Compact Nucleic acid isolation Kit (Roche Diagnostics GmbH, Mannheim, Germany). The human *IL28B* promoter polymorphism at position −3176 (rs12979860) was analysed using LightMix Kit IL28B (TIB Molbiol GmbH, Berlin, Germany).

#### Total RNA isolation and reverse transcription

The liver tissue was homogenized using a MagNA Lyser System (Roche Applied Science, Mannheim, Germany), according to the manufacturer’s instructions. Total RNA from the homogenized liver tissue was isolated using RNeasy Mini (Qiagen, Dallas, TX, USA), and total RNA from PBL using a PAXgene kit (Qiagen, Dallas, TX, USA), according to the manufacturer’s instructions. DNase treatment with the RNase-free DNase (Qiagen, Dallas, TX, USA), prior to cDNA synthesis, was carried out according to the manufacturer’s instructions. The RNA integrity was checked by agarose gel electrophoresis. First-strand cDNA was synthesized from 0,2 µg of total RNA in a final volume of 20 µl using a High-Capacity cDNA kit according to the manufacturer’s instructions (Applied Biosystems, Foster City, CA, USA).

#### RealTime HCV RNA and gene expression quantification

The HCV Primer sequences were based on data by Carriere et al. [30]. Other primers were designed using Primer 3 software (http://frodo.wi.mit.edu/primer3/. Accessed 2013 Feb 1) and synthesized by Generi Biotech (Hradec Kralove, Czech Republic) (Table 2).

**Table 2:** Primer sequences for HCV RNA, target and internal control genes.

	Forward primer	Reverse primer	Product PCR(bp)
*HCV*	GTCTAGCCATGGCGTTAGTA	CTCCCGGGGCACTCGCAAGC	246
*HMOX1*	GGGTGATAGAAGAGGCCAAGA	AGCTCCTGCAACTCCTCAAA	67
*HMOX2*	GAAGGAAGGGACCAAGGAAG	CTCCTCGAGGGCTGAGTATG	139
*BLVRA*	TCCCTCTTTGGGGAGCTTTC	GGACCCAGACTTGAAATGGAAG	180
*HPRT*	CACTGGCAAAACAATGCAGAC	GGGTCCTTTTCACCAGCAAG	92

HCV, hepatitis C virus; HMOX1, 2, heme oxygenase 1, 2; BLVRA, biliverdin reductase A; HPRT, hypoxanthine phosphoribosyltransferase.

To determine the relative expression level of all data analysis, *HPRT* expression levels were measured as internal controls. The delta cycle threshold value (Δct) was calculated from the given ct value by the formula: Δct = (ct sample – ct control). The fold change was calculated as ( = 2^−Δct^). Two reference genes (*HPRT, GAPD*) were selected as the most stable among 4 constant genes (*HPRT, GAPD, 18S RNA, UBC*) based on the analyses of 10 PBL of HCV infected patients and controls, and 10 liver samples of HCV infected patients by using geNORM 3.5 (http://medgen.ugent.be/genorm. Accessed 2009 Dec 15). Based upon similar expression levels as target genes, *HPRT* was found a more appropriate control gene, compared to *GAPD*.

qPCR was performed in 20 µl reaction volume, containing 4 µl of five-fold diluted cDNA template from completed RT reaction, 1× SYBR Green Master Mix (Applied Biosystems, Foster City, CA, USA), and 200 nM forward and reverse primers. All RT-PCR were set up in 96-well optical plates, and run on an ABI PRISM 7500 Sequence Detector System (Applied Biosystems, Foster City, CA, USA).

The cycling conditions included polymerase activation at 95°C for 10 min, followed with 40 cycles of 95°C for 15 s, and 60°C for 60 s. PCR products were subjected to a melting curve analysis. All samples were analyzed in triplicates. Linearized constructs for the PCR validation procedure were prepared using a TOPO TA Cloning Kit, according to the manufacturer’s instructions (Invitrogen, Carlsbad, CA, USA). PCR efficiencies for target and housekeeping cDNA were 97–105%.

#### HMOX activity in PBMC

Twenty µl of PBMC sonicate (2 million cells per reaction) were incubated for 15 min at 37°C in CO-free septum-sealed vials containing 20 µl of 150 µM methemalbumin and 20 µl of 4.5 mM NADPH, as previously described [31]. Blank reaction vials contained 0.1 M phosphate buffer, pH = 7.4 in place of NADPH. The reactions were terminated by adding 5 µl of 30% (w/v) sulfosalicylic acid. The amount of CO generated by the reaction and released into the vial headspace was quantified by gas chromatography (GC) with a Reduction Gas Analyzer (Trace Analytical Laboratories, now: AMETEK Process Instrument, Newark, DE, USA). HMOX activity was calculated as pmol CO/hr/10^6^ cells.

#### Statistical analysis

The data are presented as the mean ± standard deviation (SD), or median (IQ range). Differences between the studied groups (HCV vs. controls, SVR vs. treatment-failure patients) were evaluated by an unpaired t-test, or Mann-Whitney U test. The significance of the relationship between *BLVRA* expression and the treatment outcome was determined by the chi-square test. Linear regression, Pearson’s correlation and multivariate logistic regression analyses were performed using SigmaStat software, version 3.01, for the statistical analysis. For multiple logistic regression analysis following clinically relevant variables were included: *BLVRA* expression in PBL, *IL28B* gene variants, HCV RNA levels, stage of liver fibrosis, gender, hemoglobin levels and platelet number. All analyses were performed with alpha set to 0.05.

## Results

### Basal Expression of *BLVRA* in PBL of HCV Patients

**Figure 2:**
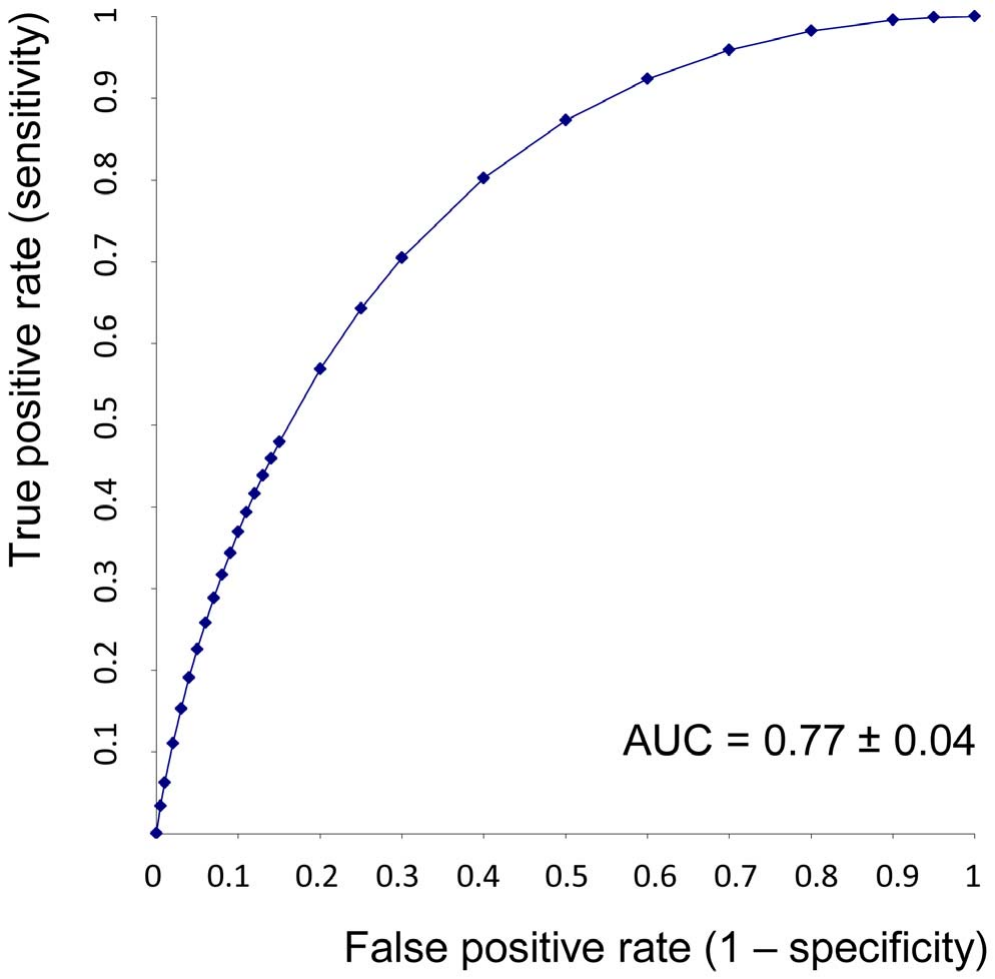
ROC curve of *BLVRA* expression in peripheral blood leukocytes of HCV infected patients. AUC, area under the ROC curve; ROC, Receiver Operating Charasteristic.

*BLVRA* expression in PBL was markedly increased in HCV-infected patients before antiviral treatment, when compared to the control group (1.68±0.68 vs. 1.28±0.36, respectively, p<0.001). Simultaneously, baseline mRNA levels of *BLVRA* were significantly higher only in patients who achieved SVR, compared to the control group (1.87±0.74 vs. 1.28±0.36, respectively, p<0.001), but not in non-SVR patients (1.32±0.34 vs. 1.28±0.36 p = 0.65). The *BLVRA* expression in PBL of relapse patients (n = 5) before antiviral treatment was substantially reduced compared to SVR patients (1.15±0.19 vs. 1.87±0.74, p<0.001), and was also decreased, although non-significantly, when compared to non-SVR patients (n = 15) (1.15±0.19 vs. 1.32±0.34, p = 0.09). Significant differences in basal *BLVRA* expression were found between SVR and non-SVR patients (Table 1). When assessing possible factors responsible for treatment response, only BLVRA expression has been found to be a strong predictor (Table 3). Exclusion of BLVRA from multivariant analysis did not have any effect on predictive value of tested variables. Based on ROC analysis (Figure 2), *BLVRA* expression predicted the treatment response with 76% sensitivity, 70% specificity (positive predictive value = 83%, negative predictive value = 61%). Since ribavirin treatment is commonly associated with hemolysis, and bilirubin overproduction might upregulate BLVRA, we assessed the relationship between ribavirin-induced drop of hemoglobin levels and *BLVRA* expression (Table 4.). However, no changes in *BLVRA* expression were detected either in 12 or 24 weeks of antiviral therapy in hemoglobin depleted patients.

**Table 3:** Multivariate logistic regression analysis of potential SVR predictors.

	OR	95% CI	P-value
***BLVRA*** ** expression PBL**	**15**	**1.05–214.2**	**0.046**
*IL28B* (rs12979860)	3.05	0.39–24	0.29
HCV RNA	1.45	0.37–5.6	0.59
Liver fibrosis	0.76	0.4–1.5	0.4
Sex	1.81	0.13–2.5	0.66
Hemoglobin	0.94	0.88–1.01	0.09
Platelets	1.01	0.94–1,03	0.18

SVR, responders; HCV, hepatitis C virus; *IL28B*, interleukin 28B; PBL, peripheral blood leukocytes; BLVRA, biliverdin reductase A; OR, odds ratio; CI, confidence interval.

*IL28B* genotype was tested as CC vs. non-CC allele carriers.

**Table 4:** The impact of ribavirin-induced anemia on *BLVRA* expression.

	anemia			Hb drop		
	Hb <100 g/L	Hb >100 g/L	*P*-value	<30 g/L	>30 g/L	*P*-value
week 12						
incidence (%)	8.6	91.4		38	62	
*BLVRA* expression	2.6±1.7	2.53±0.9	0.85	2.1±1.1	2.53±0.9	0.31
week 24						
incidence (%)	27.8	72.2		50	50	
*BLVRA* expression	2.46±1.6	2.82±0.7	0.73	2.59±1.2	2.72±0.8	0.78

BLVRA, biliverdin reductase A; HB, hemoglobin.

### Expression of BLVRA in PBL in HCV Patients during Antiviral Treatment

**Figure 3:**
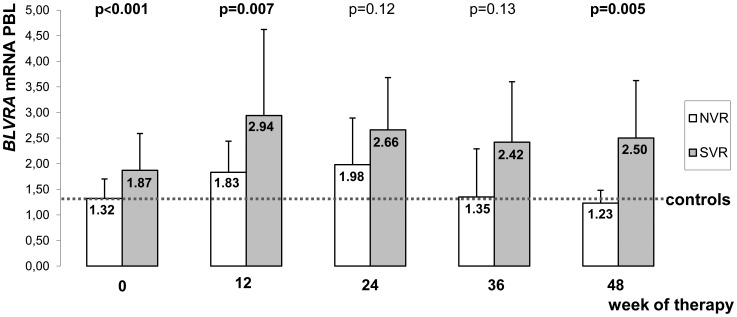
*BLVRA* expression in peripheral blood leukocytes of responders and non-SVR patients during standard antiviral therapy. *BLVRA* expression was measured the day before treatment initiation (0), and 12, 24, 36 and 48 weeks after start of the standard treatment. Data represent means and standard deviations for triplicate determinations. P-values calculated between responders and non-SVR patients. BLVRA, biliverdin reductase A; PBL, peripheral blood leukocytes; SVR = responders, NVR = non-SVR patients.

*BLVRA* expression significantly increased at weeks 12 (2.57±1.61, p = 0.0004), 24 (2.57±1.17, p = 0.0002), and 36 (2.16±1.19, p = 0.03) after initiation of standard antiviral therapy when compared to the initial levels (1.68±0.68). Significant differences in *BLVRA* mRNA levels between SVR and non-SVR patients were found at weeks 12 and 48 after treatment initiation. Similar trends were also observed at weeks 24 and 36. These differences, however, did not reached statistical significance, most likely due to the low number of subjects (Figure.3). Interestingly, *BLVRA* expression in SVR patients after withdrawal of antiviral therapy (n = 10) decreased substantially compared to *BLVRA* expression levels at week 24 (median [IQ range] 1.38 [1.2–1.6] vs. 2.52 [1.5–2.8], p = 0.03), and reached control levels (median [IQ range] 1.38 [1.2–1.6] vs. 1.28 [1–1.5], p = 0.43).

### Expression and Activity of HMOX in HCV Patients

Compared to controls, the HMOX activity in PBMC of HCV-infected patients before antiviral treatment was substantially reduced (20.6±16.3 vs 36.3±18.1 pmol CO/10^6^ cells/h, respectively, p = 0.001). Although PBL gene expression of *HMOX1* did not differ between HCV-infected patients and the control group, the *HMOX2* expression was slightly, but significantly, reduced in patients with HCV infection (4.30±1.13 vs 4.98±0.94, respectively, p = 0.001). While a significant correlation between PBL gene expression of *HMOX1, HMOX2* mRNA and HMOX activity in PBMC was only detected in control samples (r^2^ = 0.210, p = 0.007; r^2^ = 0.113, p = 0.03), this relationship was not observed in the HCV-infected patients (r^2^ = 0.040, p = 0.20; r^2^ = 0.046, p = 0.33*).* Finally, no differences in either HMOX activity or *HMOX* expression between SVR and non-SVR patients were detected.

### Correlation between *BLVRA* and *HMOX* mRNA Levels in the Liver and PBL, and HCV RNA in PBL and Liver Tissue

No significant differences in pretreatment expression of *BLVRA* in the liver were found between SVR (n = 18) and non-SVR patients (n = 4) (0.35±0.24 vs. 0.34±0.24 p = 0.97) most likely because of high variability of *BLVRA* expression in the liver compared to PBL.

*BLVRA* expression, but not that of *HMOX1/HMOX2*, in the liver and PBL of HCV-infected patients were in direct relationship (n = 13, r^2^ = 0.347, p = 0.03). No correlation was found between the mRNA levels of *HMOX1/HMOX2/BLVRA* and HCV RNA in the liver and PBL.

## Discussion

Because of the side effects and high costs of current antiviral therapy, it is very important to identify those markers that can discriminate among those patients who will respond to the standard treatment. The precise molecular mechanisms underlying the responsiveness to antiviral treatment among HCV-infected individuals have yet to be completely identified.

Enzymes of the heme catabolic pathway seem to belong to such promising markers. In fact, Zhu and coworkers [32] recently provided a plausible mechanism for the antiviral activity of HMOX1, demonstrating that the direct product of its activity, biliverdin, potently inhibits viral replication at biologically relevant concentrations in human hepatoma Huh-7.5 cells replicating HCV RNA, most likely *via* inhibition of HCV NS3/4A protease. In the current study, we prospectively investigated HMOX activity, as well as *HMOX1* expression in HCV-infected patients. Surprisingly, no difference in mRNA expression of *HMOX1* in PBL was found between therapeutically naïve HCV patients and controls, although the total HMOX activity in PBMC was significantly decreased in HCV patients before treatment, compared to the control group.

Furthermore, a correlation between the expression of *HMOX1*, *HMOX2,* and total HMOX activity was only detected in the control samples; not in the HCV-infected patients. In fact, interference of HCV with HMOX1 induction [33], reduced hepatic expression of *HMOX1* both *in vitro* and *in vivo* in HCV infection [8]; additionally, induced hepatic *HMOX1* expression *in vitro* were reported [17]. We hypothesized that *HMOX* and *BLVRA* gene expression in PBL can reflect their expression in the liver. In our study, due to unavailability of liver specimens of control subjects, correlation between the liver and PBL could be analyzed only in HCV patients. No association of *HMOX1/HMOX2* expression was found between the liver and PBL, and *HMOX1/HMOX2/BLVRA* and HCV RNA in the liver and PBL. On the other hand, expression of *BLVRA* in the liver tissue correlated with expression of *BLVRA* in PBL. The expression of *BLVRA* in PBL was higher in HCV-infected patients before antiviral therapy, compared to the control group; and subsequently increased 12, 24, and 36 weeks after initiation of standard antiviral therapy, when compared to initial levels. Most importantly, *BLVRA* expression in PBL was found to be strongly associated with response to the antiviral treatment. Recent genome-wide association studies identified strong evidence *IL28B* gene variation (rs12979860) with SVR rates in patients chronically infected with genotype 1 HCV [34], [35], [36]. Although not statistically significant, a similar trend was observed in our cohort of patients for all genotypes (p = 0.11) as well as for genotype 1 (p = 0.16). The prevalence of CC vs non CC genotypes in our group of patients corresponds to prevalence rates of chronically infected HCV patients in the Czech Republic reported recently [37]. However, it should be noted that the lack of association with other clinically important variables tested in our regression model including *IL28B* gene variation might be due to small sample size effect. Furthermore, there was a clear trend for association of upregulated baseline *BLVRA* expression in PBL of patients with favorable CC genotype as compared to both non CC (p = 0.059) and TT *IL28B* (p = 0.058) patients.

Induced *BLVRA* gene transcription in PBMC of uninfected chimpanzees in response to INF-α [38] and in INF-α treated PBMC [39] were previously reported. In accord with this data, *BLVRA* overexpression in our HCV-infected patients prior to and during antiviral treatment seems to be due to *BVLRA* upregulation by INF-α. Moreover, our results showing an association of *BLVRA* in PBL with the treatment outcome are in agreement with substantially greater global induction of IFN-stimulated genes observed in the PBMC of treatment responders [40].

This data is in accord with our observation indicating that 1) SVR patients have increased *BLVRA* expression prior initiation of therapy (likely due to endogenous interferon induced by HCV infection); 2) both SVR and non-SVR patients have increased *BLVRA* expression during antiviral therapy (likely due to exogenous interferon administered therapeutically); 3) SVR patients after withdrawal of antiviral therapy have decreased expression of *BLVRA* to control values (likely due to decreased production of endogenous interferon, since HCV, as the major stimulus, is absent). It is also important to note, that *BLVRA* expression during HCV infection and antiviral therapy is independent of ribavirin-induced hemolysis. However, our data does not provide conclusive evidence whether *BLVRA* expression is involved actively in driving the treatment response, or is just a surrogate marker for treatment responsiveness.

In conclusion, our pilot results demonstrate that patients with chronic HCV infection significantly upregulate *BLVRA* expression in PBL, closely correlating with those in liver tissue. In addition, basal *BLVRA* expression in PBL is strongly associated with response to treatment. Finally, the lack of *BLVRA* overexpression is associated with non-responsiveness to standard antiviral therapy. Nevertheless, larger prospective studies are needed to confirm our data.
